# Self-Healing, Stretchable, Biocompatible, and Conductive Alginate Hydrogels through Dynamic Covalent Bonds for Implantable Electronics

**DOI:** 10.3390/polym13071133

**Published:** 2021-04-02

**Authors:** Yeonsun Choi, Kyuha Park, Heewon Choi, Donghee Son, Mikyung Shin

**Affiliations:** 1 Department of Biomedical Engineering, Sungkyunkwan University (SKKU), Suwon 16419, Korea; choiys3854@naver.com; 2 Department of Electrical and Computer Engineering, Sungkyunkwan University (SKKU), Suwon 16419, Korea; telos6063@gmail.com (K.P.); chwchw97@gmail.com (H.C.); 3 Department of Superintelligence Engineering, Sungkyunkwan University (SKKU), Suwon 16419, Korea; 4 Department of Intelligent Precision Healthcare Convergence, Sungkyunkwan University (SKKU), Suwon 16419, Korea

**Keywords:** conductive hydrogels, alginate, self-healing, stretchability, biocompatibility, electromyogram, dynamic covalent bonds, epigallocatechin gallate

## Abstract

Implantable electronics have recently been attracting attention because of the promising advances in personalized healthcare. They can be used to diagnose and treat chronic diseases by monitoring and applying bioelectrical signals to various organs. However, there are challenges regarding the rigidity and hardness of typical electronic devices that can trigger inflammatory reactions in tissues. In an effort to improve the physicochemical properties of conventional implantable electronics, soft hydrogel-based platforms have emerged as components of implantable electronics. It is important that they meet functional criteria, such as stretchability, biocompatibility, and self-healing. Herein, plant-inspired conductive alginate hydrogels composed of “boronic acid modified alginate” and “oligomerized epigallocatechin gallate,” which are extracted from plant compounds, are proposed. The conductive hydrogels show great stretchability up to 500% and self-healing properties because of the boronic acid-cis-diol dynamic covalent bonds. In addition, as a simple strategy to increase the electrical conductivity of the hydrogels, ionically crosslinked shells with cations (e.g., sodium) were generated on the hydrogel under physiological salt conditions. This decreased the resistance of the conductive hydrogel down to 900 ohm without trading off the original properties of stretchability and self-healing. The hydrogels were used for “electrophysiological bridging” to transfer electromyographic signals in an ex vivo muscle defect model, showing a great bridging effect comparable to that of a muscle-to-muscle contact model. The use of plant-inspired ionically conductive hydrogels is a promising strategy for designing implantable and self-healable bioelectronics.

## 1. Introduction

In recent years, implantable electronics (IEs) have been developed and improved, especially in the medical field, because of their ability to improve personalized healthcare. They include biosensors, bioconductors, electrostimulated drug delivery systems, and tissue engineering for the diagnosis and treatment of chronic diseases [1]. IEs can transmit and receive bioelectronic signals from the brain, heart, or muscle, for monitoring and curing chronic diseases [2,3,4,5]. However, it is challenging to overcome the drawbacks of the hard and rigid characteristics of conventional IEs [6]. As mechanical rigidity causes acute and chronic inflammation, soft and flexible properties matching those of biological tissues are required. To develop soft IE applications, conductive hydrogels have attracted attention as a candidate substrate for IEs because hydrogels can retain abundant water molecules similar to tissues, and the moist environment can offer a consecutive ionic conductivity [7]. Nevertheless, there are many other requirements for a proper conductive hydrogel, including biocompatibility [8,9,10], stretchability [11], self-healing [12], and conductivity [13]. It is difficult to find an appropriate material with balanced electronic and mechanical properties. In addition, it is challenging to fabricate soft and flexible conductive hydrogels with multiple desirable properties.

Natural polymers, such as polysaccharides (alginate [14], chitosan [15], and hyaluronic acid [16]) and proteins (gelatin and silk) have generally been used to fabricate soft platforms such as hydrogels and aerogels [17] for medical applications because of their inherent renewability, nontoxicity, tissue compatibility [18], water solubility, biodegradability [19], microporosity [17] and moldability [20]. Due to these properties, natural polymers can be utilized as nontoxic, biocompatible conductive hydrogels that can be used for IEs, tissue scaffolds [21,22], and drug delivery systems [23,24,25]. Moreover, the structures of natural polymers can be modified with various functional groups, such as catechol, gallol, and boronic acid moieties with cis-diol, to improve the mechanical properties of hydrogels, required for various applications [26,27,28]. In addition to natural polymers, nature-derived small compounds, such as flavonoids, e.g., epigallocatechin-gallate (EGCG) [29], are also important sources of conductive hydrogels. Such compounds can be extracted from all plant and renewable resources [30]. A few studies have focused on developing a new type of conductive hydrogel consisting of nature-derived polymers/oligomers.

**Figure 1:**
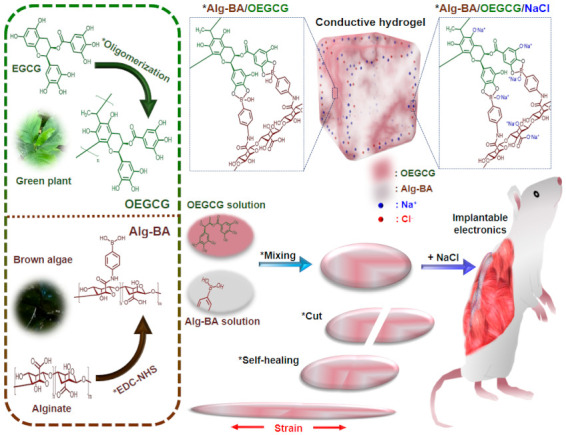
Schematic description for preparing conductive, stretchable, and self-healable boronic acid-modified alginate (Alg-BA)/oligomerized epigallocatechin-gallate (OEGCG) hydrogels via boronic acid-cis-diol reversible dynamic covalent bonds post-soaking in salt solution and their potential applications for implantable electronics. The left panel shows each chemical structure of Alg-BA or OEGCG.

In this study, a natural conductive hydrogel is proposed based on “boronic acid-modified alginate” (Alg-BA) [31] and “oligomerized epigallocatechin-gallate” (OEGCG) [32] for a soft IE platform (Figure 1). The attributes of the boronic acid-cis-diol dynamic covalent bond include stretchability and self-healing [33]. To improve the conductivity without trading off the stretchability and self-healing properties, a simple but efficient method was introduced that involves soaking in a sodium chloride solution. Furthermore, a compatibility test and electromyogram (EMG) analysis [34,35] were implemented to test the applicability of this conductive hydrogel. This strategy for fabricating a conductive hydrogel with a natural polymer and increasing the conductivity by adopting a salt solution can be useful for improving the IE platform.

## 2. Materials and Methods

### 2.1. Preparation of Alg-BA and OEGCG

3-Aminophenyl boronic acid (BA) was conjugated with the alginate (medium viscosity, Sigma-Aldrich, St. Louis, MO, USA) backbone via EDC/NHS (1-(3-Dimethylaminopropyl)-3-ethylcarbodiimide hydrochloride/N-hydroxysuccinimide) reactions, following a previous report [20]. In detail, 1 g of alginate was dissolved in 250 mL of 0.1 M MES buffer solution, and the pH was adjusted to 5. Then, 700 mg of EDC and 100 mg of NHS dissolved in 10 mL of deionized water (DW) was added dropwise to the alginate solution. Boronic acid (300 mg) dissolved in 10 mL of DW was added to the mixture solution and allowed to react for 12 h at room temperature, 25 °C. After the completion of the reaction, the resulting solution was dialyzed (molecular weight cutoff (MWCO) = 6–8 kDa) for 3 d and lyophilized.

OEGCG was synthesized using a method reported in our previous work [16]. To synthesize OEGCG, EGCG (552 mg, Healthy Origins, Morgan, PA, USA) was dissolved in a total volume of 38.652 mL of a solvent mixture of DW (33.6 mL)/dimethyl sulfoxide (4.512 mL)/acetic acid (540 μL). The oligomerization of EGCG was initiated by the addition of acetaldehyde (6 mL, Sigma-Aldrich). The reaction was continued for 48 h. After the completion of the reaction, the sample was dialyzed (MWCO = 3.5 kDa) in methanol (2 L) for 2 d and in DW (1 L) for one subsequent day. Finally, the resulting solution was lyophilized.

### 2.2. Rheological and Morphological Characterization

Alg-BA 2 *w/v*% dissolved in DW was mixed with OEGCG 10 *w/v*% dissolved in DW at a volume ratio of 5:2 to create a transparent brown hydrogel (Alg-BA/OEGCG). Sodium chloride (NaCl) was added to the Alg-BA/OEGCG hydrogel to improve the conductivity of the hydrogel for bio-electro devices (Alg-BA/OEGCG/NaCl). The sequence of mixing the three components—Alg-BA, OEGCG, and NaCl—was varied to determine a proper gelation method. First, Alg-BA/OEGCG hydrogel was soaked in a solution with various NaCl concentrations (1 *w/v*% and 2 *w/v*%). Second, OEGCG and NaCl were initially mixed, and then they were mixed with Alg-BA. Finally, OEGCG was added to a solution of Alg-BA with NaCl. The rheological properties of the hydrogels were measured using a Discovery Hybrid Rheometer 2 (TA Instrument, New Castle, DE, USA) with a 20 mm-diameter parallel plate geometry and gap size of 300 μm via frequency sweep from 0.01 to 10 Hz at 2% strain and 25 °C. For the morphological characterization of the hydrogels, Alg-BA/OEGCG or Alg-BA/OEGCG/NaCl gels were freeze-dried. The morphology of each hydrogel was analyzed by scanning electron microscopy (SEM) (JSM-7600F Schottky field emission scanning electron microscope, JEOL USA, Inc., Peabody, MA, USA).

### 2.3. In Vitro Cytocompatibility

The in vitro biocompatibility was confirmed using cell viability tests. First, L929 cell suspension (50,000 cells per well) was dispensed in a 24 well plate and preincubated at 37 °C and 5% CO_2_ for 24 h. Second, 10 mg of Alg-BA/OEGCG hydrogels with and without soaking in 1% NaCl solution were added to each cell using transwell inserts (8.0 μm pores). Cell viability was assessed 24 h after treatment using a live/dead assay (Thermo Fisher Scientific, Waltham, MA, USA). The number of live or dead cells was analyzed using ImageJ software, and the cellular viability (%) was calculated.

Additionally, the in vitro cell viability was demonstrated using a cell counting kit (CCK-3000, Dongin LS, Seoul, Korea) assay. Similar to the aforementioned live/dead assay, the hydrogels were incubated on cells using transwell inserts (24 well) for 24 h. The cell culture media (DMEM; Dulbecco′s modified Eagle′s medium) was removed, and fresh DMEM was filled in each well after washing with Dulbecco’s phosphate buffered saline (DPBS). Then, CCK-8 reagent was added to each well (10% of the media) and incubated for 2 h. The absorbance at 450 nm was detected using a SYNERGY HTX multi-mode microplate reader (BioTek, Winooski, VT, USA).

### 2.4. Resistance Strain Tests

To measure the electrical conductivity of the Alg-BA/OEGCG conductive hydrogel with different soaking conditions (NaCl 1%, and NaCl 2%), hydrogels were sculpted to a cuboid shape (0.5 × 0.5 × 2 cm^3^). Each conductive hydrogel was analyzed for resistance using a probe station (MST 5500 B, MSTECH Inc., Korea) with an LCR (Inductance/Capacitance/Resistance) meter basic support program (4284 A, Agilent Technology Inc., Santa Clara, CA, USA). Simultaneously, repeated cyclic strain tests (10 times) from 0 to 100% and increased cyclic strain tests (50% per cycle) from 50 to 500% at speeds of 20% per second and 1% per second were conducted for Alg-BA/OEGCG hydrogel with and without soaking in NaCl 1%, and NaCl 2% solution. Each resistance–strain test was performed using a step motor controller (SMC-100, Ecopia Corp., Anyang, Korea) with an automatic stretch-testing machine (Stretching Tester, Jaeil Optical System Corp., Incheon, Korea). All the experiments were performed under ambient conditions. The electrical conductivity was calculated using the following Equation (1) [35]:

***σ = L/(R × A)*** (1)

where ***σ*** is the electrical conductivity (S/cm^−1^); ***R*** is electrical resistance (Ω); ***A*** is the cross-sectional area of the hydrogels (cm^2^); and ***L*** is the length of the hydrogel (cm).

### 2.5. Self-Healing Tests

The self-healing property of the Alg-BA/OEGCG hydrogel was confirmed by cutting the surfaces and attaching the cut surfaces to each other. First, a heart-shaped hydrogel which was not soaked in 1% NaCl solution was cut and attached, and then it was pulled to both sides. The hydrogel was blended completely and pulled to both sides. After soaking in 1% NaCl solution for 1 min, the hydrogel was also cut and attached to each cut surface and pulled to both sides.

The self-healing properties of the Alg-BA/OEGCG hydrogels with and without soaking the hydrogels in NaCl solution (1% and 2%) were then assessed using a rheometer. Measurements were performed under an alternating oscillatory strain at a fixed frequency of 1 Hz. Storage and loss moduli were measured by alternating strains of 0.5% and 1000% at 3 min intervals at 1 Hz.

### 2.6. EMG Tests

To verify the performance of Alg-BA/OEGCG/NaCl conductive hydrogel as an IE platform, an ex vivo muscle defect model was set up using rat hind limb muscles (12-week-old, male) labeled as ‘distal muscle’ and ‘proximal muscle’. Two muscle tissues were freshly dissected and spaced with a distance of 4 mm and the hydrogel was filled in the space. One proximal muscle was electrically stimulated by waveform generator (Arbitrary, 1 Channel, 20 MHz, 33511B, Keysight Technologies, Inc., Santa Rosa, CA, USA). The EMG signals were recorded in another distal muscle by biosignal amplifier (Bio Amp FE231, AD instruments, Sydney, Australia) and the data acquisition device (PowerLab 8/35, AD instruments). Action potential signals were filtered on the authority of the ISEK (International Society of Electrophysiology and Kinesiology) standard (1500 Hz low-pass). As a control, a direct contact muscle-to-muscle model was prepared without a space between two muscles.

## 3. Results and Discussion

### 3.1. Formation of Alg-BA/OEGCG/NaCl Hydrogels and Their Rheological Characterization

**Figure 2:**
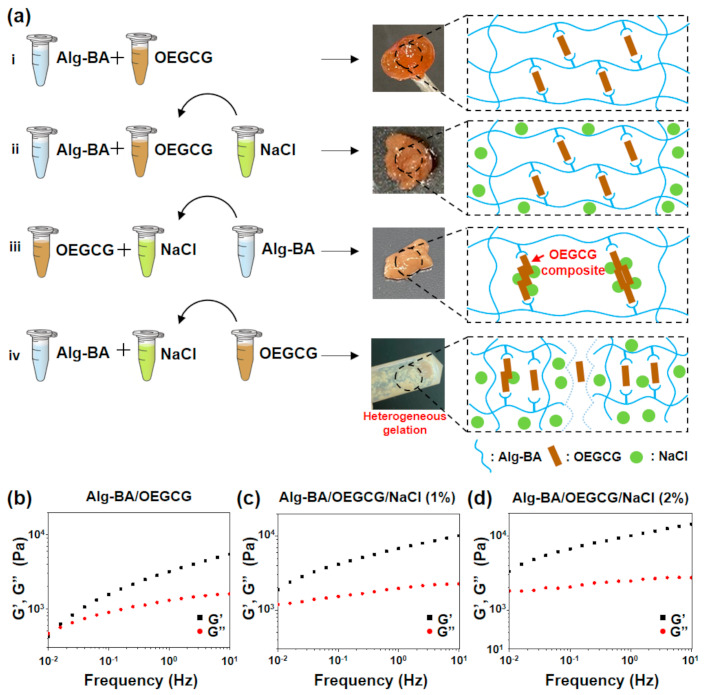
Conditions of gelation and mechanical characterization of Alg-BA/OEGCG/NaCl hydrogel. (**a**) Schematic illustration of the condition of gelation with varying sequences of mixing the components Alg-BA, OEGCG, and NaCl, and the schematic presentation of the mechanism of the bonding of components (right side), and (**b**–**d**) frequency sweep—storage (G’) and loss (G”) moduli at 2% strain, 25 °C: Alg-BA (2%)/OEGCG (**b**), Alg-BA (2%)/OEGCG/NaCl (1%) (**c**), and Alg-BA (2%)/OEGCG/NaCl (2%) (**d**).

The general approach to the formation of Alg-BA/OEGCG hydrogels is the mixing of Alg-BA and OEGCG with vigorous stirring. In an aspect of storage for further applications, the shelf life of the hydrogels might be longer than at least one month because the hydrogels contain alginate backbone which is a major polymeric network and OEGCG as a crosslinker to interconnect their network, with strong anti-oxidant and anti-microbial effects. In general, the unmodified alginate hydrogels are totally degraded in vivo within one month [24,36,37,38]. In our system, the conjugation of boronic acid onto alginate might enhance the shelf-life of unmodified alginate due to the increase in relative molecular weight and inter-crosslinking density, and OEGCG can inhibit the growth of micro-organism [39,40,41]. To improve the ionic conductivity of the hydrogels, NaCl was introduced into the Alg-BA/OEGCG hydrogels. To find an effective way of introducing NaCl into the hydrogels, the sequence of mixing the three components was varied (Figure 2a). The first hydrogel was prepared by mixing Alg-BA and OEGCG to form a brown hydrogel (Figure 2ai). After soaking in the NaCl solution, the Alg-BA/OEGCG hydrogel hardened from the surface, and the width of the hardened surface varied depending on the concentration of NaCl solution and soaking time (Figure 2aii). In the other mixing sequence, OEGCG and NaCl were initially mixed to form a blurred solution. This showed that NaCl caused OEGCG to be tangled together [42]. The OEGCG/NaCl composites were mixed with Alg-BA to form an opaque brown hydrogel (Figure 2aiii). This was different from the hydrogel soaking in NaCl solution, in that the OEGCG that combined with Alg-BA was either single or composite. Finally, the hydrogel which OEGCG was added into—the mixture solution of Alg-BA/NaCl—failed to form a homogeneous hydrogel (Figure 2aiv). Based on these results, the sequence of mixing components is important for forming a homogeneous hydrogel and determining the properties of hydrogels. NaCl ions blurred the OEGCG solution, which means that the composites formed, and the Alg-BA could couple with the OEGCG composite rather than a single OEGCG molecule. Furthermore, the presence of NaCl ions with Alg-BA hindered the coupling of Alg-BA and OEGCG. Therefore, the Alg-BA/OEGCG hydrogel was preferentially made, and it was soaked in NaCl solution to permeate through the hydrogel surface. Both Alg-BA/OEGCG and Alg-BA/OEGCG/NaCl (1%) hydrogels showed a microporous structure with the pores of approximately 200 μm (Supplementary Figure S1), which was similar to that of typical alginate hydrogels [43].

The rheological properties of the Alg-BA/OEGCG hydrogels were measured before and after soaking in NaCl solutions. The storage and loss moduli of Alg-BA/OEGCG (Figure 2b), Alg-BA/OEGCG/NaCl (1%) (Figure 2c), and Alg-BA/OEGCG/NaCl (2%) (Figure 2d) hydrogels were measured by a frequency sweep from 0.01 to 10 Hz. The results showed the dynamic covalent bond of the hydrogels, regardless of soaking in NaCl solutions. As a result, the Alg-BA/OEGCG hydrogels retained dynamic covalent bonds both before and after soaking in NaCl solutions. Therefore, the method of introducing NaCl by soaking does not change the bonds making the hydrogels. In addition, the storage moduli increased as the concentration of the NaCl solution increased from 1% (Figure 2c) to 2% (Figure 2d). Thus, the physical properties of the hydrogels could be adjusted by regulating the concentration of NaCl solutions according to the usage of the supported electronics.

### 3.2. Cytocompatibility and Biocompatibility of Alg-BA/OEGCG/NaCl Hydrogels

**Figure 3:**
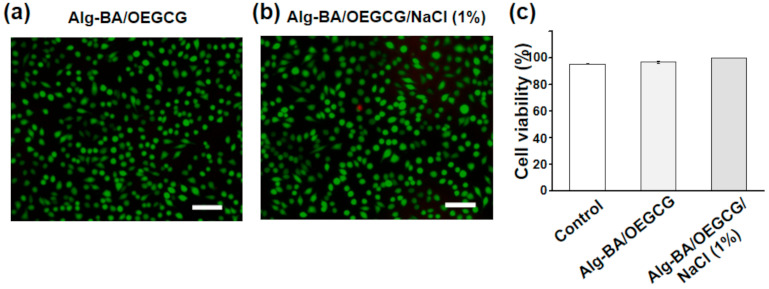
Viability of Alg-BA/OEGCG/NaCl hydrogel: (**a**,**b**) representative live/dead staining images of L929 cells after treatment with transwell Alg-BA/OEGCG (**a**) and Alg-BA/OEGCG/NaCl (1%) (**b**) after 24 h incubation at 37 °C. Living cells appear green, dead cells appear red. Scale bars: 100 μm. (**c**) Cell viability of control (95.4%), Alg-BA/OEGCG (96.9%), and Alg-BA/OEGCG/NaCl (1%) (99.7%): the error bars represent the standard deviations (*n* = 3).

To demonstrate that the Alg-BA/OEGCG/NaCl hydrogel could be used in bio-electronics without inducing toxicity, an in vitro viability test was performed using a live/dead assay. Almost all cells in both Alg-BA/OEGCG and Alg-BA/OEGCG/NaCl appeared green, indicating that almost all cells were alive, and very few cells appeared red (dead cells) (Figure 3a,b). The results showed minimal cytotoxicity from the Alg-BA/OEGCG hydrogel (>96.9% viability, Figure 3c) and Alg-BA/OEGCG/NaCl 1% hydrogel (>99.7% viability, Figure 3c). In addition, as a result of the CCK assay, the cell viability was 91.1% for Alg-BA/OEGCG hydrogels and 75.8% for Alg-BA/OEGCG/NaCl ones (Supplementary Figure S2). When NaCl was permeated to the hydrogel, cell viability was slightly decreased but still higher than 75%. Taken together, the cytocompatibility of the hydrogels with/without salt is as great as a bioelectronic material. For further discussion of potential in vivo biocompatibility, Alg-BA has been orally administered as a gastrointestinal mucoadhesive material and subcutaneously injected, showing a negligible immune response at 7 days after the injection. In addition to that, OEGCG have been considered as a non-toxic protein-drug carrier for cancer therapy [44]. Thus, the Alg-BA/OEGCG hydrogels corresponding to the mixture of non-toxic two components might exhibit good biocompatibility.

### 3.3. Electrical Properties of Alg-BA/OEGCG/NaCl Hydrogels

**Figure 4:**
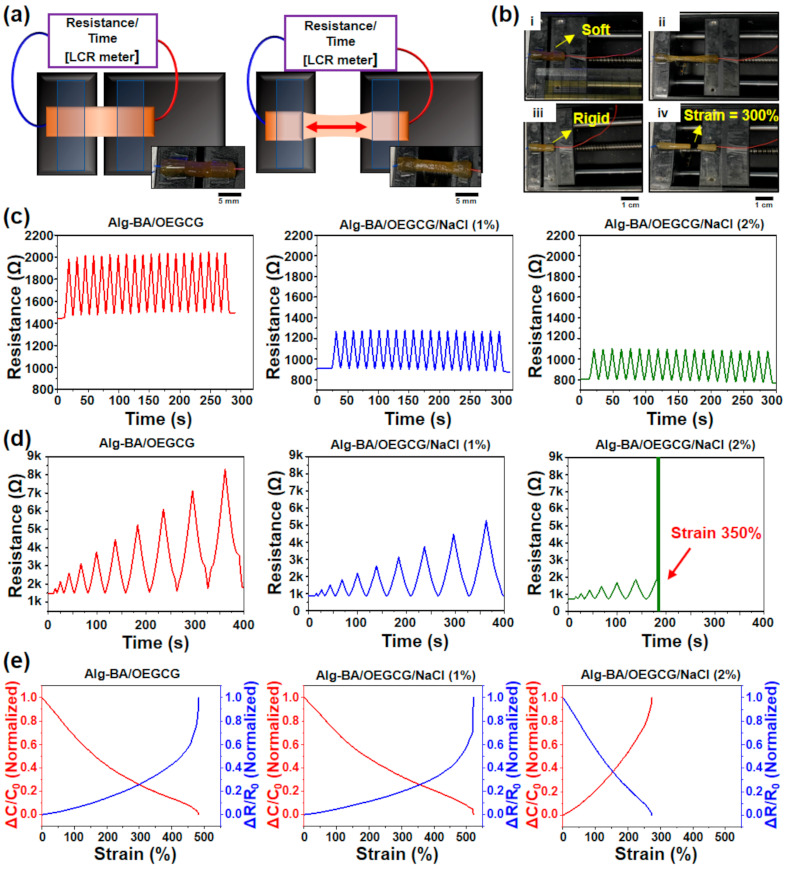
Resistance–strain cyclic test of different Alg-BA/OEGCG/NaCl conductive hydrogels to analyze the electrical-mechanical characterizations. (**a**,**b**) Illustration and photograph of resistance–strain test with automatic stretch-testing machine (LCR (Inductance/Capacitance/Resistance) meter). Alg-BA/OEGCG/NaCl was lusterless and rigid (**b**, (iii) and (iv)) compared with Alg-BA/OEGCG (**b**, (i) and (ii)) and only strained near 300%. (**c**,**d**) Resistance–strain cycle tests (10 cycles) from 0 to 100% (**c**) and strain increased strains (50% per time) from 50 to 500% (**d**). (**e**) Strain–normalized conductivity and resistance continuous strain to each conductive hydrogel—without NaCl (left), NaCl (1%) (middle), and NaCl (2%) (right). Each cyclic loading/unloading was performed continuously.

The results of the resistance–strain tests of Alg-BA/OEGCG (both without and with NaCl 1% soaking) samples showed a highest reported strain of approximately 500%, similar to previously reported stretchable hydrogels based on alginate with polyacrylic acid [45]. Repeated cyclic strains (10 cycles) from the 0 to 100% test showed a regular increase and decrease in the resistance of Alg-BA/OEGCG and Alg-BA/OEGCG/NaCl (1% and 2%) (Figure 4c). The resistance of Alg-BA/OEGCG/NaCl hydrogel was lower than that of Alg-BA/OEGCG hydrogel. The increased cyclic strains (50% per cycle) from the 50 to 500% test showed that the conditions without soaking and with soaking in the NaCl 1% solution have a similar tensile strength of approximately 500% (Supplementary Video S1). However, Alg-BA/OEGCG/NaCl (2%) decreased the maximum strain by approximately 300%. The result demonstrated that Alg-BA/OEGCG conductive hydrogels soaked in NaCl solution decreased the resistance of the hydrogel by approximately two times (1.7 to 1 kΩ). However, soaking the Alg-BA/OEGCG conductive hydrogel in NaCl (more than 2%) solution made the hydrogel rigid and lusterless because of a decrease in the strain of approximately 70% (Figure 4b,d). It is speculated that this phenomenon stems from the disturbance of the self-healing mechanism by sodium ions. The results of analyzing the normalized electrical characteristics (resistance and conductivity) per strain at a low increase support the previous cycle strain tests (Figure 4e).

### 3.4. Self-Healing Behavior of the Alg-BA/OEGCG/NaCl Hydrogels

**Figure 5:**
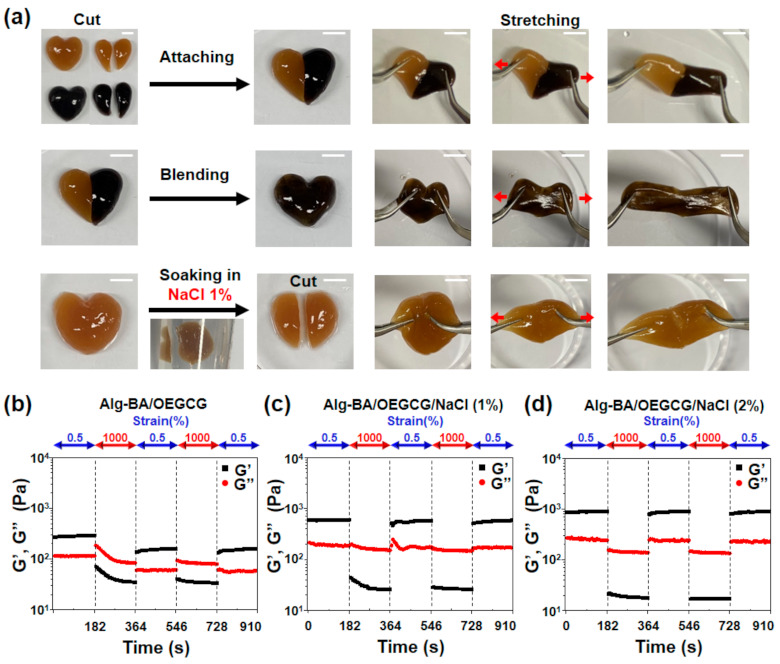
Self-healing property of the Alg-BA/OEGCG/NaCl conductive hydrogel. (**a**) Macroscopic self-healing images of Alg-BA/OEGCG hydrogel without NaCl soaking (top), completely blending without NaCl soaking (middle), and soaked in NaCl 1% solution for 1 min (bottom). (**b**–**d**) Evaluation of disruption and recovery of storage (G’) and loss (G”) moduli of hydrogels depending on an alternating strain of 0.5% and 1000%. Alg-BA (2%)/OEGCG (**b**), Alg-BA (2%)/OEGCG/NaCl (1%) (**c**), and Alg-BA (2%)/OEGCG/NaCl (2%) (**d**).

Self-healing is a key advanced property not only required for IEs but also for wearable electronics [46,47,48]. The macroscopic self-healing property of the Alg-BA/OEGCG hydrogel was confirmed by cutting and attaching cut surfaces to each other. Before soaking in 1% NaCl solution, the hydrogel stretched well and was not detached when the hydrogel was pulled to both sides (Figure 5a, top). The hydrogel blended completely, indicating the existence of self-healing properties (Figure 5a, middle). It could also stretch well to both sides. After the hydrogel was soaked in 1% NaCl solution for 1 min, it was pulled to both sides after being cut and attached again to each cut surface. It could stretch well and did not detach because NaCl did not penetrate the hydrogel due to the short time period (Figure 5a, bottom). However, when it was cut first and then soaked in NaCl solution, the attached surface combined with NaCl; hence, it did not attach properly (Supplementary Figure S3).

The self-healing properties of the Alg-BA/OEGCG hydrogels were evaluated by measuring the storage modulus and loss modulus. They were measured under alternating oscillatory strain between 0.5% and 1000% at 3 min intervals at a fixed frequency of 1 Hz. The hydrogels with and without soaking in NaCl solution recovered the storage moduli at 0.5% strain after 1000% strain (Figure 5b–d).

### 3.5. Ex Vivo Electromyographic Performance of the Alg-BA/OEGCG/NaCl Hydrogels

**Figure 6:**
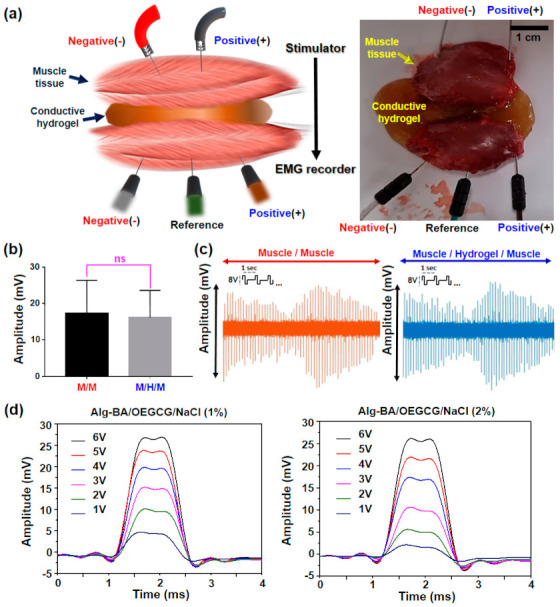
Electrophysiological bridging effect of conductive hydrogel in ex vivo muscle defect model. (**a**) Schematic illustration (left) and photograph (right) of experimental setup for the EMG recording in two muscles spaced with the hydrogels. For EMG recording, three electrodes (anode, cathode, and reference) were utilized. (**b**) Average values of maximum EMG amplitude in muscle-to-muscle (M/M) or muscle-to-hydrogels-muscle (M/H/M) model during 8 V electrical stimulation (*n* = 3). (**c**) Pattern shapes of EMG signals in each M/M and the M/H/M model. (**d**) Comparison of the EMG amplitude monitored on the muscle spaced with the hydrogel prepared in NaCl 1% (left) or 2% (right) varying from 1 to 6 V electrical stimulation.

For the evaluation of the electrophysiological bridging effect of the Alg-BA/OEGCG/NaCl hydrogels, we used an ex vivo muscle defect model based on our previous study (Figure 6a) [35]. The proximal muscle lump was electrically stimulated using a pulse with 8 V amplitude at a frequency of 1 Hz, and the action potential signal in the distal muscle lump through the Alg-BA/OEGCG/NaCl conductive hydrogel was recorded with a 200 kHz sampling frequency and an amplitude range of ±50 mV using three penetrating electrodes (Supplementary Video S2). As a result, The EMG amplitude and signal pattern recorded in the distal muscle had no significant difference in both muscle-to-muscle (M/M) direct contact model (~17 mV) and muscle-to-hydrogel-to-muscle (M/H/M) model (~16 mV) (Figure 6b,c) [49,50,51], which indicates the electrophysiological support function of these hydrogels to effectively transfer the action potential of stimulated muscle tissue to recorded tissue. Interestingly, the hydrogels prepared with a different salt concentration (NaCl 1% or 2%) showed similar efficacy to transfer the action potential (Figure 6d). Considering the previous results from resistance–strain tests (Figure 4), the hydrogel with 1% NaCl might be optimal for further biomedical applications because of their great stretchability and self-healing properties.

## 4. Conclusions

Alg-BA/OEGCG conductive hydrogel that can be utilized as an IE substrate was developed. Alg-BA/OEGCG hydrogel was formed by reversible dynamic covalent bonds between the boronic acid moiety of Alg-BA and cis-diol of the gallol moiety of OEGCG and NaCl was introduced to increase the conductivity. The blending procedure of components (Alg-BA, OEGCG and NaCl) is crucial for forming a dynamic covalent hydrogel (the sequence NaCl being added in the final order in the soaking method is an ideal case). Rheological characterizations of the hydrogels showed increasing moduli when Alg-BA/OEGCG hydrogels were soaked in NaCl solutions with higher concentrations. The conductive hydrogels are biocompatible (≥75% cell viability). Resistance–strain cyclic tests resulted in a resistance decrease of approximately 1.7 to 1 kΩ when the Alg-BA/OEGCG conductive hydrogel was soaked in NaCl solution. In contrast, soaking in more than 2% NaCl solution decreased the maximum strain by approximately 70%. Furthermore, Alg-BA/OEGCG hydrogel segments could be attached together and be completely self-healing and stretchable, even when soaked in NaCl solution. Finally, the EMG recording demonstrated an Alg-BA/OEGCG/NaCl conductive hydrogel as a role of an electrical interconnector. When it was placed between the parallel rat hindlimb muscle tissues, the amplitude and pattern of muscle–hydrogel–muscle signal showed a similar amplitude compared with muscle-to-muscle. Both the results of the EMG and resistance strain tests proved that “NaCl 1%” conductive hydrogel was an optimal platform to Alg-BA/OEGCG hydrogel (more than 2% makes hydrogel rigid with no advance). Thus, the proposed Alg-BA/OEGCG/NaCl hydrogel showed a satisfactory performance as a biocompatible conductive hydrogel which can be utilized in IEs. It can also be promising in many fabrications because of its biocompatibility on a basis of nature-derived compounds.

## Data Availability

The data presented in this study are available in the article.

## Supplementary Materials

The following are available online at https://www.mdpi.com/article/10.3390/polym13071133/s1, Supplementary Figure S1: Self-healing property of Alg-BA/OEGCG/NaCl hydrogel. Supplementary Video S1: Resistance–strain tests, Supplementary Video S2: EMG recording.
