# Supplementary material for: Self-Healing, Stretchable, Biocompatible, and Conductive Alginate Hydrogels through Dynamic Covalent Bonds for Implantable Electronics
Source: Polymers (Basel). 2021 Apr 2;13(7):1133. doi: 10.3390/polym13071133 (PMC8038184; doi:10.3390/polym13071133)
Supplement: Supplementary file 1 [file polymers-13-01133-s001.zip › Choi et al_Supplementary Materials_Polymers_2021-1164533.docx]

Supplementary Materials

Self-healing, Stretchable, Biocompatible and Conductive Alginate Hydrogels through Dynamic Covalent Bonds for Implantable Electronics

Yeonsun Choi ^1†^, Kyuha Park^2†^, Heewon Choi^2†,^ Donghee Son^2,3*^, and Mikyung Shin^1,4*^

^1^ Department of Biomedical Engineering, Sungkyunkwan University (SKKU), Suwon 16419, Republic of Korea

^2^ Department of Electrical and Computer Engineering, Sungkyunkwan University (SKKU), Suwon 16419, Republic of Korea

^3^ Department of Superintelligence Engineering, Sungkyunkwan University (SKKU), Suwon 16419, Republic of Korea

^4^ Department of Intelligent Precision Healthcare Convergence, Sungkyunkwan University (SKKU), Suwon 16419, Republic of Korea

†These authors contributed equally to this work.

* Correspondence: mikyungshin@g.skku.edu, daniel3600@g.skku.edu

Supplementary Figure S1-S3

The legend of Supplementary Movie S1, 2

**Supplementary Figure S1**


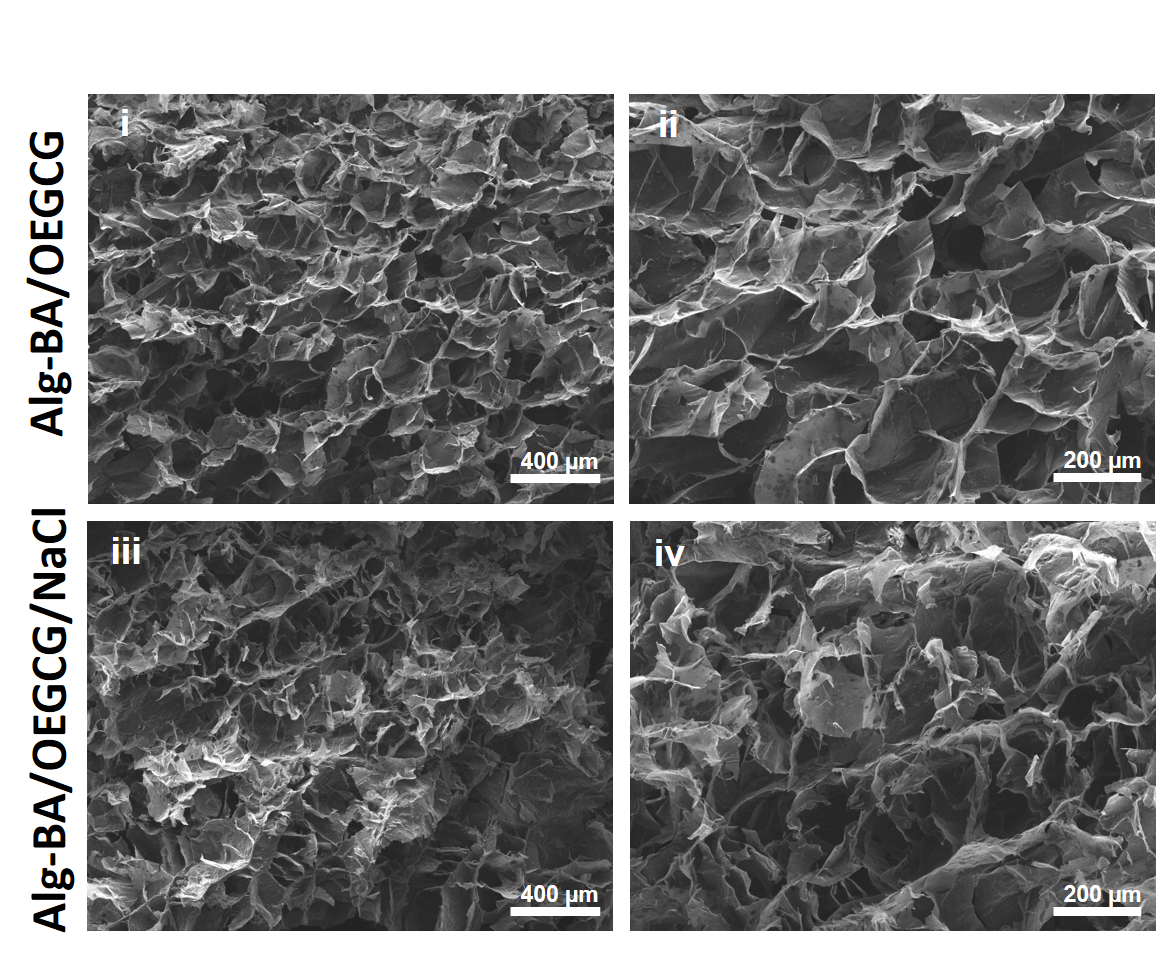


**Supplementary Figure S1.** SEM images of the lyophilized Alg-BA/OEGCG hydrogel (i for 50 magnification, ii for 100 magnification) or the Alg-BA/OEGCG/NaCl (1%) hydrogel (iii, iv).

**Supplementary Figure S2**


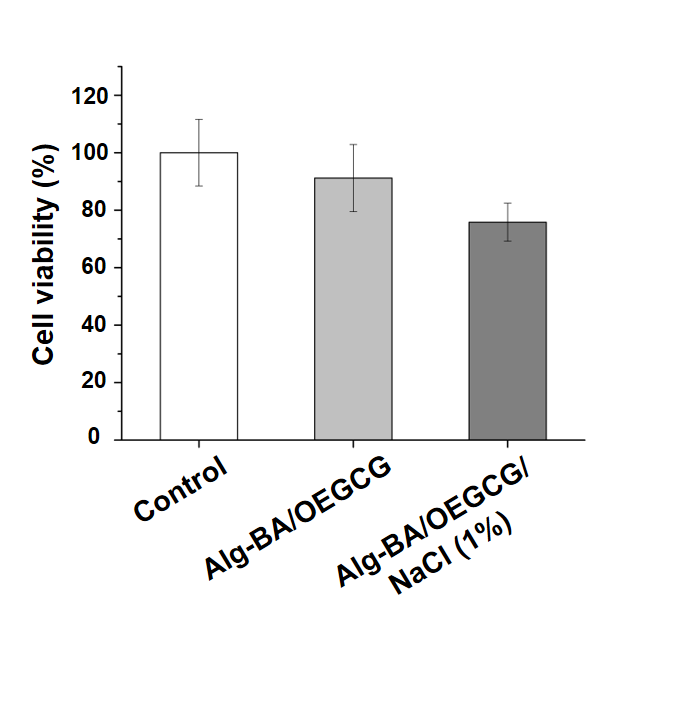


**Supplementary Figure S2.** *In vitro* cell viability of Alg-BA/OEGCG hydrogel with/without NaCl (1%) by CCK assay. A control without treatment of the hydrogel.

**Supplementary Figure S3**


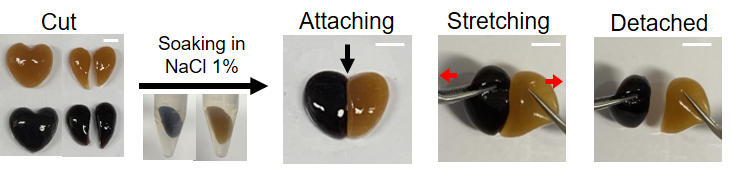


**Supplementary Figure S3.** Self-healing property of Alg-BA/OEGCG/NaCl hydrogel.

A figure shows the example of impossibility of self-healing tests of Alg-BA/OEGCG/NaCl hydrogel. The Alg-BA/OEGCG hydrogel was preferentially cut and each piece of hydrogel was soaked in NaCl 1% solution. Then, they were attached together and stretched on both sides. It was detached when it was stretched because the faces of hydrogel that were attached were associated with the NaCl ions.

**Supplementary Video S1.** Resistance-strain tests.

A movie shows the actual setup of resistance-strain tests. Fixed Alg-BA/OEGCG conductive hydrogel on the automatic stretch-testing machine was stretched while recording the variation of resistance on both ends of the hydrogel through the probe station connected to single channel electrodes (blue and red wires). The test was proceeded by cyclic strains up to 5 times.

**Supplementary Video S2.** EMG recording.

A movie shows exact demonstration of EMG recording by Alg-BA/OEGCG/NaCl 1% conductive hydrogel. The hydrogel placed between the parallel muscle lumps and acting as an electrical inter-connector. Proximal muscle was stimulated by 1 Hz square wave of 8 V and action potential had recorded from distal muscle.
